# The U-shaped association between normal-range serum bile acid levels and prognosis of Coronary Heart Disease

**DOI:** 10.3389/fendo.2025.1730403

**Published:** 2026-01-12

**Authors:** Yu Xu, Ning Ding, Rui Hua, Yue Wu, Ting Li, Zuyi Yuan

**Affiliations:** 1 Department of Cardiovascular Medicine, The First Affiliated Hospital of Xi’an Jiaotong University, Xi’an, Shaanxi, China; 2 Key Laboratory of Molecular Cardiology of Shaanxi Province, Xi’an Jiaotong University, Xi’an, Shaanxi, China; 3 Key Laboratory of Environment and Genes Related to Diseases, Ministry of Education, Xi’an Jiaotong University, Xi’an, Shaanxi, China

**Keywords:** bile acids and salts, biomarkers, coronary disease, prognosis, risk factors

## Abstract

**Background:**

Previous studies have shown that serum total bile acid levels are associated with both the presence of Coronary Heart Disease and the severity of coronary artery lesions. Coronary Heart Disease progression frequently involves an imbalance in bile acid metabolism. This study aims to evaluate the predictive value of total bile acid levels for the occurrence of major adverse cardiovascular events in patients with Coronary Heart Disease.

**Method:**

A total of 2,974 patients with Coronary Heart Disease who met the inclusion criteria were categorized into four groups based on the quartiles of their total bile acid levels: Group 1 (0.2-1.9 µmol/L, n = 760), Group 2 (1.9-3.1 µmol/L, n = 758), Group 3 (3.1-5.1 µmol/L, n = 741), and Group 4 (5.1-10 µmol/L, n = 715). Survival differences among the groups were evaluated using Kaplan-Meier curves. Multivariate Cox regression analysis was performed to assessed their associations. Restricted cubic spline models were employed to investigate potential nonlinear relationships. Subgroup interaction and incremental predictive value analyses were additionally performed.

**Results:**

Over a median follow-up period of 75 months, Group 2 exhibited a significantly lower incidence of major adverse cardiovascular events compared to the other groups. Kaplan-Meier survival analyses also demonstrated that participants in Group 2 exhibited significantly higher cumulative survival rates free of major adverse cardiovascular events compared to those in the other groups. Multivariate Cox regression revealed that both higher and lower total bile acid levels were associated with an increased risk of major adverse cardiovascular events (HRQ1 = 1.91, p<0.001; HRQ4 = 1.62, p<0.001). Furthermore, Restricted cubic spline analysis indicated a significant nonlinear, U-shaped relationship between total bile acid levels and major adverse cardiovascular events, which was statistically significant in males but not in females. Moreover, risk model analysis demonstrated that adding TBA to a conventional model significantly improved risk discrimination and reclassification.

**Conclusion:**

Total bile acid levels have predictive value for major adverse cardiovascular events in Coronary Heart Disease patients. This finding substantiates the predictive value of total bile acid as an effective risk stratification tool for patients with Coronary Heart Disease after hospital discharge.

## Introduction

Coronary Heart Disease is the leading cause of death and disability worldwide, with its health and economic burden continuously increasing. By 2050, it is projected to become the primary cause of disability-adjusted life years globally (1, 2). In the United States, approximately one-quarter of all deaths are attributed to CHD, which imposes an economic burden exceeding 50 billion US dollars, ranking first among cardiovascular disease-related costs (3, 4). Currently, in addition to using comorbidities and risk factors for long-term prognostic risk scoring, certain hematological markers are also employed as independent predictors of MACE in CHD patients. For example, the triglyceride-glucose index is considered a valuable marker for risk stratification and prognosis in patients with acute coronary syndrome (5).

Bile acids are a group of endogenous metabolites synthesized from cholesterol in the liver and subsequently modified by the intestinal microbiota. They play a crucial role in regulating lipid digestion, cholesterol metabolism, and the composition of the intestinal microbiota by binding to FXR and TGR5 receptors and activating their associated signaling pathways (6–8). Several studies have reported associations between serum TBA levels and the presence of CHD and acute coronary syndrome, as well as the severity of coronary artery lesions (9, 10). Previous research has established that bile acids, which are metabolites of cholesterol, are closely associated with the onset and progression of CHD (11). Moreover, clinical evidence suggests that patients with familial hypercholesterolemia and impaired bile acid synthesis exhibit a significantly elevated risk of developing CHD (12).In postmenopausal women, low TBA levels are independently associated with the occurrence of CHD (13). Additionally, bile acid excretion levels have been observed to be significantly lower in patients with CHD compared to those without the disease (14–16). It is worth noting that elevated TBA levels may exert cardiotoxic effects and contribute to progressive cardiomyopathy (17). In clinical trials, bile acid chelators have been shown to reduce low-density lipoprotein cholesterol and high-sensitivity C-reactive protein levels, increase the diameter of coronary artery lumen, thereby preventing the development of atherosclerosis (18–20). Inhibition of the intestinal FXR-SMPD3 axis has been shown to improve atherosclerosis (21), while activation of TGR5 can inhibit atherosclerosis by reducing macrophage inflammation and lipid accumulation (22). These findings indicate that bile acid metabolism is dysregulated in patients with CHD and that bile acid levels serve as important predictors of cardiovascular events.

However, it remains unclear whether the reference range of TBA levels have predictive value for the long-term prognosis of the CHD patients. This study aims to investigate the association between fasting serum TBA levels within the reference range and the incidence of MACE events in CHD patients, thereby aiming to validate their utility as a novel clinical biomarker.

## Method

### Study design and participants

This was a single-center cohort study with a retrospectively identified population and prospective follow-up. The sample size was determined by the consecutive enrollment of all eligible patients during the study period. This approach prioritizes representativeness and minimizes selection bias.

From July 2015 to January 2020, 3358 patients with CHD were consecutively admitted to the first affiliated hospital of Xi’an Jiaotong University. We conducted a follow-up with these patients five years after their discharge, and the follow-up was completed in January 2025.

Inclusion criteria were: (1) age ≥18 years; (2) availability of TBA test results; and (3) TBA levels within the normal reference range (0–10 μmol/L). The exclusion criteria were as follows: (1) age under 18 years; (2) pregnancy; (3) malignant tumors; (4) severe renal insufficiency or dialysis; (5) severe liver insufficiency, defined as serum alanine aminotransferase (ALT) levels exceeding three times the upper limit of normal; (6) biliary obstructive diseases or use of bile acid–chelating drugs; (7) missing TBA test results or TBA levels above the reference range; and (8) loss to follow-up.

**Figure 1:**
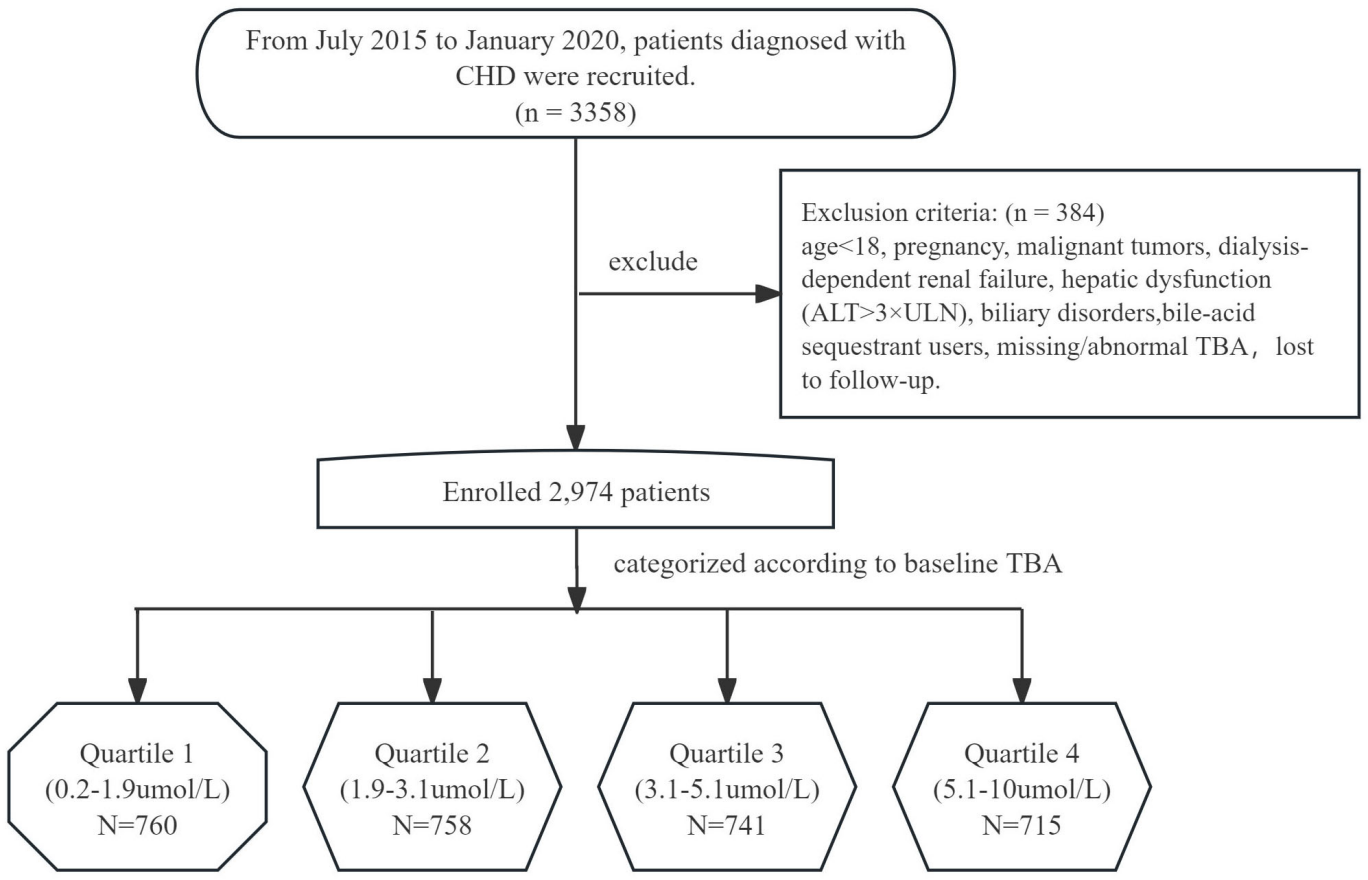
Flow diagram of study participants. CHD, Coronary Heart Disease; ALT, alanine aminotransferase; TBA, total bile acids.

Missing baseline TBA data occurred in less than 1% of patients and were considered missing at random; therefore, no imputation was performed. A total of 2,974 patients met these criteria and were included. These patients were then divided into four groups based on the quartiles of their TBA levels (**Figure 1**).

### Data collection instruments

The detailed demographic, clinical, drug, hematologic, and angiographic data were retrospectively extracted from the hospital’s comprehensive electronic medical record and case management systems. A standardized data collection sheet was designed to systematically capture all relevant variables. The demographic variables included respondent age, sex, and education status. History of comorbidities (including cancer, diabetes, hypertension, dyslipidemia), smoking and alcohol consumption status, and relevant medication use were collected via patient self-reporting.

Follow-up Instrument: The main method of follow-up involved structured telephone interviews carried out by trained research staff who were blinded to the patients’ baseline TBA levels. A standardized questionnaire was used during the interviews to systematically inquire about the occurrence of pre-defined MACE, including all-cause mortality, non-fatal Myocardial Infarction, Stroke, and unplanned revascularization. The time to event occurrence was calculated from the date of TBA measurement to the end of follow-up or to the date of death or MACE.

Validity and Reliability: The validity of the data is supported by its origin from routine clinical practice within a tertiary care center. Laboratory data are highly reliable due to standardized automated analyzers and strict internal quality control. Our hospital has a follow-up office and corresponding regulations in place to ensure the quality of follow-up services. CHD was diagnosed based on coronary angiography results, which were evaluated by at least two experienced interventional cardiologists. Diagnosis required at least 50% stenosis in one or more major coronary arteries. To ensure data extraction consistency, two trained researchers independently reviewed a random sample of 100 records.

### Procedures

This study involved two separate data collection phases: a retrospective baseline phase and a prospective follow-up phase. The procedure for the follow-up phase is as follows.

Procedure of Accessing the Participants: Contact information was obtained from the hospital’s EMR. Participants were contacted during working hours. Up to three attempts were made on different days to reach each participant or their family members.

Setting and Response to the Questionnaire: The interviews were conducted in a quiet, dedicated office space to ensure privacy and minimize background noise. The research staff used a standardized electronic questionnaire to systematically record responses in real-time.

Gathering Completed Questionnaires: Completed electronic questionnaire records were saved and aggregated within a secure, password-protected database after each successful telephone call immediately. This process ensured immediate and lossless data collection.

Verification and Adjudication: All reported events from the telephone interviews were independently adjudicated by two experienced cardiologists according to pre-established criteria.

### Ethical considerations

Patients were treated according to standard clinical guidelines. This study was conducted in accordance with the Declaration of Helsinki and was approved by the Ethics Committee of the First Affiliated Hospital of Xi’an Jiaotong University (Approval Code: XJTU1AF2019LSK-G-2; Date of Approval: February 25, 2019). All participants voluntarily took part and provided written informed consent. All data were kept strictly confidential, and the anonymity of participants was preserved throughout the study.

### Data analysis

The analysis was conducted using SPSS version 26 and R software. Continuous variables with a normal distribution were expressed as mean ± standard deviation (SD); otherwise, they were presented as median (interquartile range, IQR). Categorical variables were reported as frequency (percentage). For group comparisons, the chi-square test or Fisher’s exact test was used for categorical variables, as appropriate for frequency data. Analysis of variance (ANOVA) was applied to normally distributed continuous variables, while the Kruskal-Wallis test was used for non-normally distributed continuous variables, selected based on data distribution. Multiple comparisons with Bonferroni correction were performed for variables showing significant differences among the four groups to adjust for the increased risk of false discoveries. Univariate and multivariate Cox regression models were employed to analyze the risk of composite cardiovascular adverse events and all-cause mortality in patients with CHD across different TBA levels. Survival curves were constructed using the Kaplan-Meier method, and group comparisons were conducted with the log-rank test. To investigate the potential nonlinear relationship between TBA and outcome events, restricted cubic splines (RCS) were employed. Based on the data distribution, four knots were placed at the 5th, 35th, 65th, and 95th percentiles of TBA. Adjustments were made for age, sex, BMI, and other confounding factors, and sex-stratified analyses were performed. To evaluate TBA’s incremental predictive value, we compared a baseline Cox model with conventional risk factors to an extended model including TBA quartiles, assessing improvements in discrimination (C-statistic, AUC) and reclassification (NRI, IDI). A p-value less than 0.05 was considered statistically significant for all tests.

## Results

### Clinical character

The baseline characteristics of the study cohort are presented in **Table 1**. A total of 2,974 patients were included in the statistical analyses and stratified into four groups based on the quartiles of total bile acid (TBA) levels: Q1 (TBA 0.2-1.9 µmol/L, n = 760), Q2 (TBA 1.9-3.1 µmol/L, n = 758), Q3 (TBA 3.1-5.1 µmol/L, n = 741), and Q4 (TBA 5.1-10 µmol/L, n = 715). Significant differences were observed among these groups with respect to age, sex, systolic blood pressure, low-density lipoprotein cholesterol, triglycerides, white blood cell count, creatinine, thyroid-stimulating hormone, aspartate aminotransferase, and alanine aminotransferase (*p<*0.05). In contrast, no statistically significant differences were found in medical history, medication adherence, or other baseline laboratory parameters (*p*>0.05) (**Table 1**).

**Table 1:** Comparison of baseline characteristics among the four groups.

Characteristics	Quartile 1	Quartile 2	Quartile3	Quartile 4	P-value
	n=760	n=758	n=741	n=715	
Age, year	59.7 ± 10.7	60.5 ± 10.6	62.2 ± 10.2	62.3 ± 9.8	**<0.001**
Female (%)	172 (22.6)	194 (25.6)	211 (28.5)	216 (30.2)	**0.006**
Smoking (%)	350 (46.1)	341 (45.0)	335 (45.2)	353 (49.4)	0.307
Hypertension (%)	205 (27.0)	222 (29.3)	232 (31.3)	220 (30.8)	0.255
Diabetes Mellitus (%)	200 (26.3)	210 (27.7)	215 (29.0)	209 (29.2)	0.570
BMI, kg/m2	22.3 ± 7.4	22.6 ± 7.1	22.4 ± 7.4	22.7 ± 7.0	0.879
Previous Stroke (%)	45 [5.9)	43 (5.7)	39 (5.3)	37 (5.2)	0.913
Renal Insufficiency (%)	32 (4.2)	39 (5.1)	25 (3.4)	36 (5.0)	0.312
Heart Failure (%)	35 (4.6)	29 (3.8)	33 (4.5)	28 (3.9)	0.841
Atrial Fibrillation (%)	23 (3.0)	20 (2.6)	21 (2.8)	19 (2.7)	0.965
PCI (%)	90 (11.8)	85 (11.2)	83 (11.2)	76 (10.6)	0.909
Glucose, mmol/L	6.2 [5.0, 8.2]	6.2 [5.0, 8.2]	6.1 [5.0, 8.0]	6.1 [5.0, 8.1]	0.782
Total Cholesterol mmol/L	3.8 [3.2, 4.6]	3.8 [3.2, 4.4]	3.8 [3.2, 4.4]	3.8 [3.2, 4.5]	0.170
LDL-cholesterol mmol/L	2.3 [1.7, 2.9]	2.2 [1.7, 2.7]	2.2 [1.6,2.7]	2.2 [1.6,2.7]	**0.019**
HDL-cholesterol mmol/L	1.0 [0.8, 1.1]	0.9 [0.8, 1.1]	0.9 [0.8, 1.1]	1.0 [0.8, 1.1]	0.110
Triglycerides mmol/L	1.4 [1.0, 1.9]	1.4 [1.0, 2.1]	1.4 [1.0, 1.9]	1.4 [1.0, 2.0]	**0.002**
HGB, g/L	136.0 [121.3, 148.0]	137.0 [120.0, 149.0]	136.0 [120.0, 146.0]	136.0 [122.0, 146.0]	0.358
WBC,10^9/L	7.1 [5.4, 9.8]	6.5 [5.1, 8.1]	6.3 [4.9, 7.6]	6.0 [4.7, 7.8]	**<0.001**
GFR ml/min/1.73m^2^	79.2 [0.0,110.8]	75.2 [0.0, 110.0]	82.2 [0.0, 113.2]	86.3 [0.0, 116.9]	0.124
Scr, umol/L	64.0 [53.0, 75.0]	62.8 [52.0, 73.2]	63.0 [54.0, 73.0]	61.2 [52.0, 72.0)	**0.027**
TSH, mIU/L	1.4 [0.8, 2.4]	1.6 [1.0, 2.8]	1.8 [1.1, 3.0]	2.0 [1.2,3.0]	**<0.001**
AST U/L	29.2 [20.0, 72.0]	25.0 19.0, 39.0]	23.1 [19.0, 33.3]	23.8 [19.0, 32.0]	**<0.001**
ALT U/L	29.0 [17.3,53.7]	25.6 [16.4, 41.9]	22.3 [15.0, 33.4]	22.6 [16.1, 36.4]	**<0.001**
Aspirin (%)	751 (98.9)	749 (98.8)	735 (99.2)	707 (98.9)	0.878
Plavix (%)	540 (71.1)	535 (70.6)	538 (72.6)	520 (72.7)	0.730
β-blocker (%)	680 (89.5)	683 (90.1)	663 (89.5)	648 (90.6)	0.860
ACEI/ARB (%)	580 (76.3)	568 (74.9)	545 (73.5)	537 (75.1)	0.673
Statin (%)	743 (97.8)	735 (97.0)	723 (97.6)	698 (97.6)	0.766

Significant values are in bold.

BMI, body mass index; PCI, percutaneous coronary intervention; LDL, low-density lipoprotein; HDL, high-density lipoprotein; HGB, hemoglobin; WBC, white blood cell; GFR, glomerular filtration rate; Scr, serum creatinine; TSH, thyroid stimulating hormone; ALT, alanine aminotransferase; AST, aspartate aminotransferase.

### Clinical outcomes

The median follow-up time for the four patient groups was 75 months (interquartile range: 67–84 months). During this period, a total of 653 MACE events occurred, representing 22% of the cohort. These events included 205 (27%) in the Q1 group, 116 (15.3%) in the Q2 group, 141 (21.3%) in the Q3 group, and 174 (24.3%) in the Q4 group. The MACE event rate was lowest in the Q2 group, and this difference was statistically significant. Notably, within the normal range, both excessively high and low TBA levels were associated with an increased incidence of MACE. Similarly, the incidence of Myocardial Infarction and revascularization was also lowest in the Q2 group, with statistically significant differences. No significant differences were observed among the four groups in rates of all-cause mortality, Heart Failure, or Stroke (**Table 2**).

**Table 2:** Outcomes of patients stratified by TBA quartiles.

Outcomes	Total (n=2974)	Quartile 1 (n=760)	Quartile 2 (n=758)	Quartile 3 (n=741)	Quartile 4 (n=715)	P
MACE (%)	653 (22)	205 (27.0)	116 (15.3)	158 (21.3)	174 (24.3)	**<0.001**
All-Cause Mortality	189 (6.4)	56 (7.4)	38 (5.0)	46 (6.2)	49 (6.9)	0.269
Myocardial Infarction	102 (3.4)	43 (5.7)	19 (2.5)	17 (2.3)	23 (3.2)	**<0.001**
Heart Failure	190 (6.4)	54 (7.1)	34 (4.5)	49 (6.6)	53 (7.4)	0.087
Revascularization	231 (7.8)	79 (10.4)	35 (4.6)	54 (7.3)	63 (8.8)	**<0.001**
Stroke	91 (3.1)	28 (3.7)	19 (2.5)	18 (2.4)	26 (3.6)	0.311

Categorical variables were presented as number (percentage). P values were calculated using Chi-square test to compare differences in outcomes between different TBA quartiles.

TBA, total bile acids; MACE, major adverse cardiovascular events. Significant values are in bold (according to the formula for Bonferroni-corrected p-values, the original p-value must be less than 0.0167 to achieve significance).

**Figure 2:**
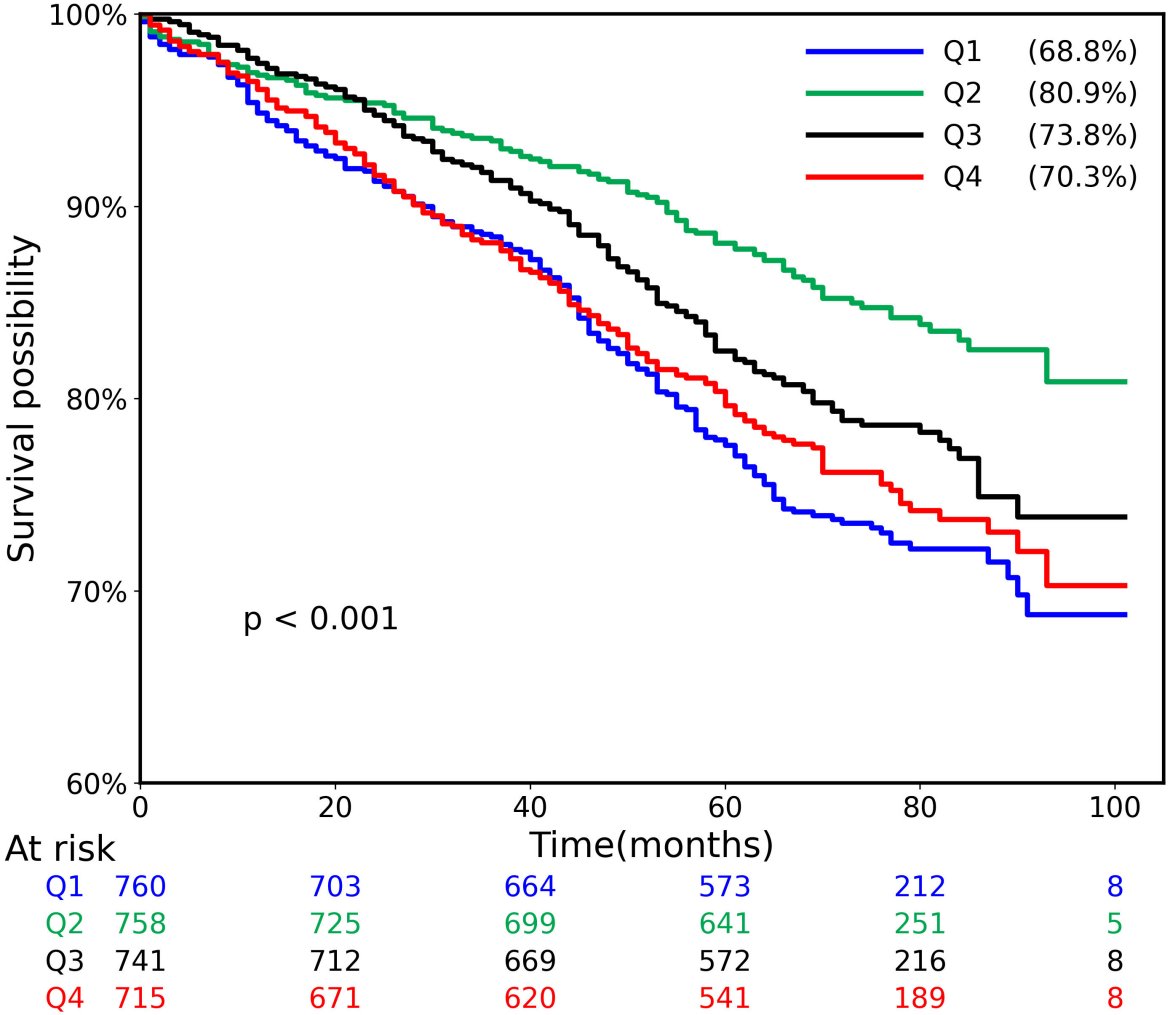
Kaplan-Meier survival curves without complex cardiovascular events among the four groups (log-rank, P < 0.001).

As shown in the Kaplan-Meier plot (**Figure 2**), the Q2 group demonstrated a better prognosis compared to the other groups, exhibiting a higher MACE-free survival rate than the Q1, Q3, and Q4 groups (log-rank test, *p* < 0.001).

### Independent association of TBA levels with MACE

Cox regression analysis was performed to compare the risk of MACE events among four patient groups (**Table 3**). After adjusting for age, gender, smoking status, hypertension, diabetes, body mass index (BMI), triglycerides (TG), low-density lipoprotein (LDL), serum creatinine (Scr), alanine aminotransferase (ALT), aspartate aminotransferase (AST), thyroid-stimulating hormone (TSH), and white blood cell count (WBC), the analysis revealed that the Q2 group had the lowest risk of MACE events (HR_Q1_ = 1.910, 95% CI: 1.517-2.404, *P<*0.001; HR_Q4_ = 1.615, 95% CI: 1.275-2.046, *P* < 0.001). Additionally, both low and high TBA levels were associated with an increased risk of revascularization (HR_Q1_ = 2.524, 95% CI: 1.687-3.777, *P* < 0.001; HR_Q4_ = 1.830, 95% CI: 1.208-2.771, *P* = 0.004). Furthermore, lower TBA levels were linked to a higher risk of Myocardial Infarction (HR_Q1_ = 2.316, 95% CI: 1.337-4.011, *P* = 0.003).

**Table 3:** Cox regression analysis of the association between TBA and endpoint events.

Endpoint events	Quartile 1 (N = 760)		Quartile 2 (N = 758)	Quartile 3 (N = 741)		Quartile 4 (N = 715)	
	HR (95%CI)	P	Reference	HR (95%CI)	P	HR (95%CI)	P
MACE							
Model1	1.892 (1.506-2.376)	**<0.001**	Ref	1.449 (1.140-1.841)	**0.002**	1.705 (1.348-2.157)	**<0.001**
Model2	1.954 (1.556-2.454)	**<0.001**	Ref	1.374 (1.081-1.747)	**0.009**	1.616 (1.277-2.045)	**<0.001**
Model3	1.910 (1.517-2.404)	**<0.001**	Ref	1.339 (1.053-1.704)	**0.017**	1.615 (1.275-2.046)	**<0.001**
Myocardial Infarction							
Model1	2.291 (1.335-3.931)	**0.003**	Ref	0.924 (0.480-1.778)	0.814	1.306 (0.712-2.399)	0.389
Model2	2.325 (1.354-3.990)	**0.002**	Ref	0.898 (0.467-1.729)	0.748	1.271 (0.692-2.336)	0.439
Model3	2.316 (1.337-4.011)	**0.003**	Ref	0.888 (0.461-1.713)	0.724	1.268 (0.689-2.334)	0.446
Revascularization							
Model1	2.322 (1.560-3.457)	**<0.001**	Ref	1.625 (1.062-2.486)	0.025	1.996 (1.320-3.017)	**0.001**
Model2	2.369 (1.591-3.527)	**<0.001**	Ref	1.573 (1.028-2.408)	0.037	1.935 (1.279-2.926)	**0.002**
Model3	2.524 (1.687-3.777)	**<0.001**	Ref	1.518 (0.991-2.326)	0.055	1.830 (1.208-2.771)	**0.004**

Significant values are in bold (According to the formula for Bonferroni-corrected p-values, the original p-value must be less than 0.0167 to achieve significance).

Model1: adjust none. Model2: adjust age, gender.

Model3: adjust age, gender, BMI, hypertension, LDL, TG, WBC, Scr, TSH, ALT, AST.

TBA, total bile acids; MACE, major adverse cardiovascular events; Ref, Reference; HR, hazard ratio; CI, confidence interval.

### The dose-response relationship between TBA levels and MACE

**Figure 3:**
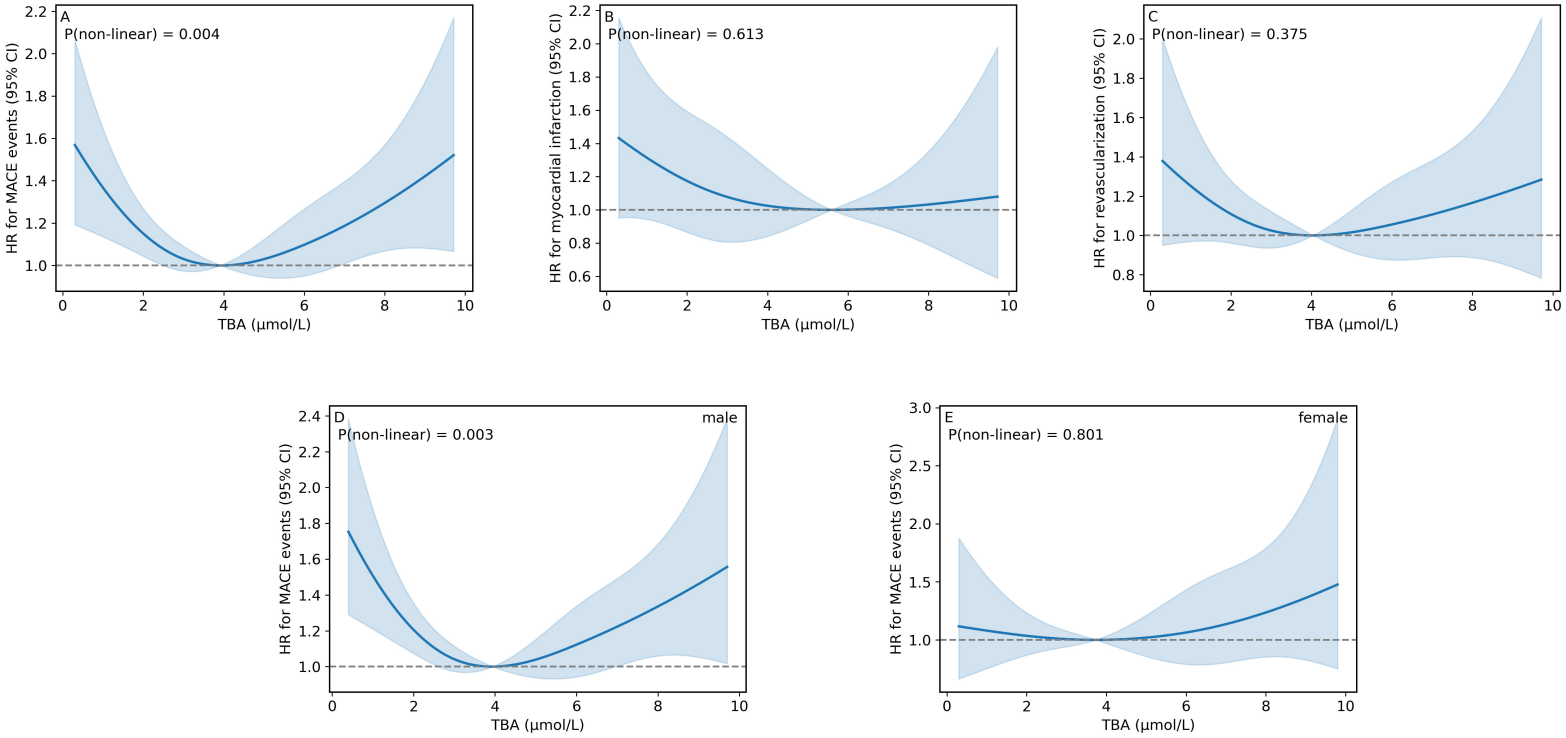
Dose–response relationships between TBA and cardiovascular outcomes. **(A)** Nonlinear association between TBA and MACE. **(B)** Association between TBA and Myocardial Infarction. **(C)** Association between TBA and Revascularization. **(D)** Nonlinear association between TBA and MACE in male participants. **(E)** Association between TBA and MACE in female participants.Hazard ratios (solid lines) with 95% confidence intervals (shaded areas) were derived from restricted cubic spline analyses after adjusting for age, gender, BMI, hypertension, LDL, TG, WBC, Scr, TSH, ALT, AST.TBA, total bile acids; MACE, major adverse cardiovascular events.

Using a restricted cubic spline (RCS) model to analyze the nonlinear relationship between TBA levels and MACE events (after adjusting for confounding factors such as age, gender, hypertension, and diabetes), we found a U-shaped association between serum TBA levels within the reference range and the incidence of MACE. The risk of MACE initially decreased as TBA levels increased, then began to rise again when TBA exceeded 3.98 μmol/L (**Figure 3A**). Although the overall interaction was significant (P for overall interaction = 0.016), gender-specific analysis revealed that the U-shaped association between TBA and MACE was statistically significant only in the male subgroup, with no significant association observed in female participants (**Table 4**; **Figures 3D, E**).

**Table 4:** Subgroup analysis for the association of TBA with MACE.

Variable	Quartile 1		Quartile 2	Quartile 3		Quartile 4	
	HR (95%CI)	P	Reference	HR (95%CI)	P	HR (95%CI)	P
Total	1.910 (1.517-2.404)	**<0.001**	Ref	1.339 (1.053-1.704)	**0.017**	1.615 (1.275-2.046)	**<0.001**
Male	2.384 (1.807-3.146)	**<0.001**	Ref	1.607 (1.196-2.158)	**0.002**	1.830 (1.364-2.455)	**<0.001**
Female	1.033 (0.663-1.610)	0.884	Ref	0.866 (0.565-1.325)	0.507	1.197 (0.800-1.790)	<0.381
P^a^	**0.002**			0.034		0.135	
P^b^	**0.016**						

HRs were adjusted for age, gender, BMI, hypertension, LDL, TG, WBC, Scr, TSH, ALT, AST.

P^a^; P value for interaction term.

P^b^; Overall P value for interaction between sex and TBA levels.

Significant values are in bold (according to the formula for Bonferroni-corrected p-values, the original p-value must be less than 0.0167 to achieve significance).

Both higher and lower TBA levels within the reference range were associated with an increased rate of revascularization; however, this nonlinear relationship did not reach statistical significance (**Figure 3C**).

### The incremental predictive value of TBA for MACE

The addition of TBA quartiles to a conventional risk model demonstrated a statistically significant improvement in the prediction of MACE (**Table 5**). Specifically, the extended model including TBA showed improved discrimination compared to the baseline model, with the Harrell’s C-statistic increasing from 0.604 (95% CI, 0.603–0.605) to 0.623 (95% CI, 0.622–0.624). Consistently, the 5-year time-dependent area under the curve (AUC) for MACE prediction increased from 0.618 (95% CI, 0.590–0.646) to 0.639 (95% CI, 0.612–0.667), representing an absolute increase (ΔAUC) of 0.021. Furthermore, the model exhibited significant improvement in risk reclassification, with a category-free net reclassification improvement (NRI) of 0.218 (95% CI, 0.122–0.314) and an integrated discrimination improvement (IDI) of 0.009 (95% CI, 0.005–0.013).

**Table 5:** Incremental value of TBA quartiles for MACE risk prediction.

Measure	Baseline clinical model	Baseline model + TBA
Discrimination		
Harrell’s C (95% CI)	0.604 (0.603–0.605)	0.623 (0.622–0.624)
5-year AUC (95% CI)	0.618 (0.590–0.646)	0.639 (0.612–0.667)
Reclassification		
NRI (95% CI)	–	0.218 (0.122–0.314)
IDI (95% CI)	–	0.009 (0.005–0.013)

The baseline model comprised age, gender, BMI, hypertension, LDL, TG, WBC, Scr, TSH, ALT, AST.

NRI and IDI reflect the improvement achieved by adding TBA quartiles to the baseline model.

CI, confidence interval; Harrell’s C, Harrell’s concordance statistic; AUC, area under the curve; NRI, net reclassification improvement; IDI, integrated discrimination improvement.

## Discussion

The principal finding of our study is that, within the reference range, TBA levels exhibit a U-shaped association with the risk of major adverse cardiovascular events in patients with Coronary Heart Disease, which was statistically significant in males but not in females. This association remained independent of traditional risk factors, establishing TBA as a novel and independent prognostic biomarker. To our knowledge, this is the first large-scale cohort study to investigate this non-linear relationship between physiological TBA concentrations and the prognosis of CHD. This study overcomes the traditional limitation of focusing solely on cardiovascular risk associated with pathological bile acid levels.

The association between serum TBA levels and CHD has been limited and contradictory. Our discovery of a U-shaped relationship resolves these discrepancies, showing that both low and high physiological TBA levels increase cardiovascular risk, unifying previous conflicting findings into a coherent pathophysiological model. Some studies link lower TBA levels to increased CHD risk. A 20-year follow-up identified reduced bile acid production as an independent risk factor (15), and a case-control study reported significantly lower TBA levels in postmenopausal women with CHD (13) - consistent with the ascending limb of our U-curve. This association may reflect insufficient activation of cardio-protective pathways mediated by specific bile acid components. For example, ursodeoxycholic acid can promote the dissolution of cholesterol crystals, inhibit the formation of atherosclerotic plaques, and facilitate plaque regression (23). Additionally, deoxycholic acid exerts cardiovascular protective effects by inhibiting platelet activation, reducing thrombosis formation, and alleviating ischemia-reperfusion (I/R) heart injury (24). These findings indicate that insufficient physiological TBA levels may serve as an independent risk factor for CHD, revealing a potential mechanism by which low bile acid levels increase cardiovascular risk. Conversely, the right side of our U-shaped curve incorporates prior evidence of increased CHD risk at elevated TBA concentrations. Studies in asymptomatic individuals have shown that coronary artery stenosis severity increases with higher serum TBA quartiles (25). Similarly, Zeng X et al. observed a gradual increase in MACE risk when TBA exceeded reference values in patients undergoing chronic total occlusion percutaneous coronary intervention (26). These apparent discrepancies in the literature likely reflect methodological variations—including differences in diagnostic criteria, population characteristics, and a predominance of cross-sectional designs that preclude longitudinal inference. Crucially, most prior studies did not systematically examine TBA levels strictly within the physiological range, nor did they account for potential non-linear associations. Our study expands the scope to the entire CHD patient population, with a median follow-up time of up to 75 months, focusing on the predictive value of TBA levels within the physiological concentration range for MACE events. We found that both excessively high and low TBA levels within the reference range increase the risk of MACE and revascularization in CAD patients. This suggests that incorporating TBA into existing risk assessment models can identify high-risk individuals overlooked by traditional indicators and help establish the optimal target range for TBA, providing a basis for personalized treatment. Furthermore, dynamic monitoring of TBA can assess the efficacy of lipid-lowering therapies.

There are a few reasons for the prognostic influence of TBA levels in patients with CHD in this study. Some studies suggest that the activation of bile acids and their receptors may exert anti-inflammatory effects during the progression of atherosclerosis (27, 28). Dual activation of the bile acid receptors FXR and TGR5 has been shown to reduce circulating lipid levels and inflammation via the protein kinase A/nuclear factor-κB signaling pathway (29). Additionally, research indicates that bile acids can induce the expression of adhesion molecules in endothelial cells by activating reactive oxygen species, NF-κB, and p38 (30), while the bile acid receptor TGR5 inhibits platelet activation and thrombosis (31). Furthermore, bile acids reduce triglycerides via the pathway involving FXR, SHP, and SREBP-1c, thereby mediating their anti-atherosclerotic effect (32). These findings suggest that TBA may play a significant role in the progression of CHD. Further research is necessary to elucidate these mechanisms.

This study found that gender significantly influenced the prognostic association between TBA and MACE, with the U-shaped association being statistically significant in men but not in women. This may result from the lower prevalence of CHD and fewer MACE events in women, reducing statistical power, or gender-specific physiological factors such as sex hormones affecting bile acid metabolism or cardiovascular protection. Therefore, TBA’s clinical value as a biomarker may be clearer in men, but larger studies are needed to confirm its relevance in women.

The strengths of our study include the consecutive recruitment of patients based on coronary angiography over five years, which reduces misdiagnosis, and the use of a uniform methodology in a single-center setting, which ensures consistent measurement of TBA and other clinical indicators and minimizes technical errors. Our research has several limitations. First, this is a single-center study involving Chinese patients with CHD. Therefore, caution is warranted when generalizing the results to other ethnicities and populations. Multi-center, multi-ethnic studies are needed to validate our findings and clarify the underlying mechanisms. Second, the sample size was determined by consecutive enrollment over a fixed period, not by a prospective calculation, this design naturally resulted in a smaller female subgroup due to the lower population prevalence of CHD in women, which may have obscured the predictive value of TBA for MACE in female CHD patients. Future studies should include larger cohorts to verify these findings. Third, this was an observational study, our results could be influenced by unmeasured confounders. Finally, our research focused solely on fasting bile acid levels, further investigation is necessary to elucidate the roles and specific mechanisms of individual bile acid components.

Our study establishes serum TBA as an independent prognostic biomarker for CHD. The U-shaped association underscores a potential optimal range for cardiovascular health. Integrating TBA into clinical risk assessment could enable earlier identification of high-risk patients and inform personalized strategies. This proposition is further substantiated by our risk model analysis, confirming TBA’s role as a useful incremental risk stratification tool. Future research should define the precise therapeutic window for TBA and clarify its underlying mechanisms in CHD progression.

## Conclusion

This study demonstrated that, within the physiological reference range, TBA levels exhibited a U-shaped association with the occurrence of MACE in patients with CHD. These findings highlight the potential utility of TBA as a novel biomarker for risk stratification in CHD, providing a basis for individualized patient management.

## Data Availability

The original contributions presented in the study are included in the article/supplementary material. Further inquiries can be directed to the corresponding authors.
